# *Notes from the Field*: Illicit Benzodiazepines Detected in Patients Evaluated in Emergency Departments for Suspected Opioid Overdose — Four States, October 6, 2020–March 9, 2021

**DOI:** 10.15585/mmwr.mm7034a4

**Published:** 2021-08-27

**Authors:** Kim Aldy, Desiree Mustaquim, Sharan Campleman, Alison Meyn, Stephanie Abston, Alex Krotulski, Barry Logan, Matthew R. Gladden, Adrienne Hughes, Alexandra Amaducci, Joshua Shulman, Evan Schwarz, Paul Wax, Jeffrey Brent, Alex Manini, the Toxicology Investigators Consortium Fentalog Study Group

**Affiliations:** ^1^American College of Medical Toxicology, Phoenix, Arizona; ^2^The University of Texas Southwestern Medical Center, Dallas, Texas; ^3^National Center for Injury Prevention and Control, CDC; ^4^Center for Forensic Science Research and Education at the Frederic Rieders Family Foundation, Willow Grove, Pennsylvania; ^5^Toxicology Department, NMS Labs, Horsham, Pennsylvania; ^6^Oregon Health & Science University Emergency Medicine, Portland, Oregon; ^7^Lehigh Valley Health Network, Allentown, Pennsylvania; ^8^University of Pittsburgh School of Medicine, Pittsburgh, Pennsylvania; ^9^University of Washington School of Medicine, Seattle, Washington; ^10^University of Colorado Denver School of Medicine, Aurora, Colorado; ^11^Icahn School of Medicine at Mount Sinai, New York, New York.

Illicit benzodiazepines are emerging drugs of abuse that are unlawfully manufactured in laboratories and have clinical side effects and toxicity that are not well understood. Although prescription and illicit benzodiazepines are structurally similar (*1*), illicit benzodiazepines can have different pharmacological properties; this contributes to concerns about their potential potency and clinical implications (*1*,*2*). Simultaneous exposure to both illicit benzodiazepines and opioids increases overdose risk (*3*). Although naloxone will reverse opioid overdose symptoms, it does not reverse overdoses resulting from nonopioid drugs. Therefore, in cases of co-exposure to opioids and benzodiazepines, including illicit benzodiazepines, symptoms of benzodiazepine intoxication (e.g., profound sedation) are unaffected by naloxone, leading to risk for respiratory failure or death (*1*). Rapid increases in the forensic and clinical detection of illicit benzodiazepines during 2020 have raised concerns about the drug’s role in overdoses, but clinical descriptions of overdoses caused by illicit benzodiazepine co-exposure are limited (*4*–*6*). This report describes the detection of illicit benzodiazepine co-exposures among patients treated in emergency departments (EDs) with suspected opioid overdoses in selected states.

The Toxicology Investigators Consortium (ToxIC) Fentalog Study Group is conducting a study of patients aged >18 years evaluated in an ED following a suspected opioid overdose. Comprehensive toxicologic testing was performed on residual biologic samples via liquid chromatography quadrupole time-of-flight mass spectrometry for the presence of approximately 900 psychoactive substances, including 33 illicit benzodiazepines and metabolites. Additional case information was obtained through chart review.* This activity was reviewed by CDC and was conducted consistently with applicable federal law and CDC policy.^†^

During October 6, 2020–March 9, 2021, 141 biologic samples^§^ were analyzed from five clinical sites in four states (Missouri, New York, Oregon, and Pennsylvania).^¶^ The presence of illicit benzodiazepines was identified in 21 (14.9%) patients (Table); the substances identified included clonazolam (11 patients; 52.4%), etizolam (10; 47.6%), and flubromazolam (two; 9.5% [co-identified in patients with etizolam]). Among the 21 patients with illicit benzodiazepines detected, 12 (57.1%) were from Pennsylvania, six (28.6%) from Oregon, two (9.5%) from Missouri, and one (4.8%) from New York. Etizolam was confirmed in New York, Oregon, and Pennsylvania, and flubromazolam only in Oregon. At least one opioid was identified in 20 cases (95.2%), including methadone in 12 (60.0%). Either methamphetamine, amphetamine, or both were detected in 11 (52.4%) patients.

**TABLE:** Detection of illicit benzodiazepines and opioids, initial naloxone administration, and outcomes among patients with suspected opioid overdose (N = 21) — Toxicology Investigators Consortium Fentalog Study Group, four states, October 6, 2020–March 9, 2021

Characteristic	No. (%) of patients
Illicit benzodiazepines detected	
Clonazolam	11 (52.4)
Etizolam	10 (47.6)
Flubromazolam*	2 (9.5)
Opioids detected	
Co-detected opioids^†^	20 (95.2)
Methadone	12 (60.0)
Fentanyl	6 (30.0)
Heroin	4 (20.0)
Codeine	2 (10.0)
Para-fluorofentanyl	2 (10.0)
Buprenorphine	1 (5.0)
Acetyl fentanyl	1 (5.0)
Medical course and outcome	
Naloxone administration^§^	16 (76.2)
Only 1 dose administered	9 (56.3)
≥2 doses administered	7 (43.8)
Known naloxone indication^¶^	15 (71.4)
Depressed level of consciousness	9 (60.0)
Respiratory depression	7 (46.7)
Decreased oxygenation	3 (20.0)
Decreased carbon dioxide expiration	2 (13.3)
Known naloxone response**	13 (61.9)
Improved level of consciousness	6 (46.2)
Increased respiratory rate	4 (30.8)
Improved oxygenation	1 (7.7)
Precipitated withdrawal^††^	1 (7.7)
No response	5 (38.5)
Respiratory and cardiac intervention	
Endotracheal intubation/Mechanical ventilation	1 (4.8)
Cardiopulmonary resuscitation	1 (4.8)
Hospital course	
Discharge from emergency department	17 (81.0)
Admission to hospital floor	3 (14.3)
Admission to intensive care unit	1 (4.8)
Disposition	
Discharged without sequelae	18 (85.7)
Transferred to higher level of care	1 (4.8)
Transferred to substance use treatment	1 (4.8)
Left against medical advice	1 (4.8)
Died	0 (—)

* Flubromazolam was only detected in two of the cases that also included etizolam.

^†^ At least one opioid was identified in 20 cases. More than one opioid might be noted for a given case. The percentages of specific opioids are calculated based on these 20 cases.

^§^ The percentages of number of doses of naloxone are calculated based on 16 cases with naloxone administration. Initial naloxone dose was administered either outside of the hospital (by emergency medical services in 10 cases, by bystanders in two cases, and unknown in one case) or in the hospital (three cases).

^¶^ Indications for initial dose of naloxone were known in 15 of 21 total cases. The percentages of naloxone indication categories are calculated based on these 15 cases. More than one indication might be noted for a given case.

** Response to initial dose of naloxone was known in 13 of 16 naloxone administrations (81.3%). More than one response might be noted for a given case. The percentages of clinical response categories are calculated based on these 13 cases.

^††^ Precipitated withdrawal is medication-induced withdrawal that can cause particularly intense symptoms, including agitation, nausea/vomiting, and muscle aches and pains, among other withdrawal symptoms.

The mean patient age was 39 years (range = 25–63 years), and more than three quarters of patients (16; 76.2%) were men. The most commonly reported reason for opioid use was to induce euphoria (10; 47.6%), followed by use to prevent withdrawal (four; 19.0%). Naloxone was administered to 16 (76.2%) patients to reverse opioid overdose. In 15 cases for which the indication for naloxone administration was known, the most common indication was depressed consciousness (nine patients), followed by respiratory depression (seven patients). Of 13 patients for whom the response to naloxone was known, five showed no improvement after the first dose of naloxone. One patient, whose level of consciousness improved after the first dose, subsequently required 9 naloxone doses and ultimately received a naloxone infusion.

This report documents concerning co-exposure to both opioids and illicit benzodiazepines among patients evaluated for suspected opioid overdose from multiple geographically diverse U.S. EDs. Despite the fact that the sample was limited, this report’s findings align with recent increases in the supply of illicit benzodiazepines in the United States (*4*–*6*). Even though the majority of these patients were discharged without apparent sequelae, in approximately one third of cases where response to naloxone was known, patients with simultaneous exposure to both opioids and illicit benzodiazepines did not respond to naloxone. Although other factors might be involved, such as naloxone dose or administration technique, the opioid effects among these patients might have been reversed; however, these patients possibly experienced additional sedative effects from illicit benzodiazepines. The widespread use of community naloxone programs highlights the importance of calling emergency medical services after administering naloxone, because patients with co-exposure might require additional medical care. The growing use of illicit benzodiazepines requires a better understanding of the synergistic toxicity when these drugs are used along with opioids. Raising awareness among clinical, public safety, and community partners about dangers associated with the use of illicit benzodiazepines, including co-use with opioids, is critical.

## Footnotes

* The ToxIC Fentalog Study Group is conducting this study from 2020-2025. Data presented are part of an ongoing effort to assess the role and prevalence of novel substances in participating regions throughout the duration of this project. Chart review was performed via the patient's electronic medical record at the site by the medical toxicologist, the trained research assistant, or both. Reason for exposure was documented in the patients initial ED notes.

§ All suspected opioid overdose cases from participating sites that met inclusion criteria were included in the study. Inclusion criteria were met for any patient aged >18 years with a suspected opioid overdose that had a leftover blood specimen for analysis. Exclusion criteria for cases were any cases in persons with trauma or burns, or if the patient was a prisoner, pregnant, or both.

¶ Pennsylvania had two study sites.
